# The effect of microbial metabolites from colonic protein fermentation on bacteria‐induced cytokine production in dendritic cells

**DOI:** 10.1002/biof.70007

**Published:** 2025-02-24

**Authors:** Zhuqing Xie, Danny Blichfeldt Eriksen, Peter Riber Johnsen, Dennis Sandris Nielsen, Hanne Frøkiær

**Affiliations:** ^1^ Department of Food Science, Faculty of Science University of Copenhagen Frederiksberg C Denmark; ^2^ Department of Veterinary and Animal Sciences, Faculty of Health and Medical Sciences University of Copenhagen Frederiksberg C Denmark

**Keywords:** cytokine production, dendritic cells, microbial stimuli, protein fermentation metabolites, short‐chain fatty acids

## Abstract

Compared to the well‐defined immune‐modulating effect of butyrate, the understanding of the effect of other protein fermentation metabolites is limited. This study aimed to investigate the impact of protein‐derived metabolites (valerate, branched‐chain fatty acids, ammonium, phenol, p‐Cresol, indole, and hydrogen sulfide) on cytokine production in murine bone marrow‐derived dendritic cells (BMDCs) stimulated with lipopolysaccharides (LPS), *Lactobacillus acidophilus* NCFM, or *Staphylococcus aureus* USA300. Some of the metabolites, but not the short‐chain fatty acids (SCFAs), strongly affected cell viability. After short‐term treatment and depending on the microbial stimulus, SCFAs affected the cytokine profile similarly but weaker than butyrate, as reflected by inhibition of IL‐12p70 and IL‐10 but enhanced IL‐23 (LPS and *S. aureus* USA300) and IL‐1β production. Compared to butyrate, valerate exhibited a weaker and slower effect on cytokine expression. Two‐day treatment with valerate and butyrate resulted in similar effects, that is, LPS‐induced IL‐12 abrogation and IL‐10 enhancement, increased aryl hydrocarbon receptor (*Ahr*) expression, and after LPS stimulation, increased expression of dual specificity phosphatase 1 (*Dusp1*). In conclusion, SCFAs exhibited low toxicity and modulated microbially stimulated BMDCs. Valerate and butyrate showed the strongest effect, which was dependent on the specific microbial stimulation and the course of the SCFA treatment. Our work adds knowledge regarding the role of protein‐derived metabolites from gut bacterial fermentation on the immune system.

## Graphical abstract

The effect of protein‐derived metabolites (butyrate, valerate, branched‐chain fatty acids, ammonium, phenol, p‐Cresol, indole, and hydrogen sulfide) on cytokine production in murine bone marrow‐derived dendritic cells stimulated with lipopolysaccharides (LPS), *Lactobacillus acidophilus* NCFM, or *Staphylococcus aureus* USA300 was investigated. The fatty acids stood out among these compounds, and valerate in specific holds activities resembling but not identical to butyrate.

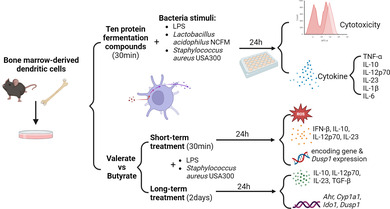

**Abbreviations**
7‐AAD: 7‐aminoactinomycin D
AHR: aryl hydrocarbon receptor
B: butyrate
BCFAs: branched‐fatty acids
BMDCs: bone marrow‐derived dendritic cells
CFU: colony‐forming unit
DCs: dendritic cells
Dusp1: dual specificity phosphatase 1
ELISA: enzyme‐linked immunosorbent assay
FBS: fetal bovine serum
GM‐CSF: granulocyte‐macrophage colony stimulating factor
GPR: G‐protein‐coupled receptors
H2S: hydrogen sulfide
HDACs: histone deacetylase
IB: iso‐butyrate
IDO1: indoleamine 2,3‐dioxygenase 1
IFN‐β: interferon beta
IL: interleukin
IN: indole
IV: iso‐valerate
MB: 2‐methylbutyrate
MFI: mean fluorescent intensity
N: ammonium chloride
NADPH: nicotinamide adenine dinucleotide phosphate
PBS: phosphate‐buffered saline
pC: p‐Cresol
PH: phenol
pre‐B: pre‐treated with butyrate
pre‐V: pre‐treated with valerate
qPCR: quantitative real‐time polymerase chain reaction
ROS: reactive oxygen species
RPMI: Roswell Park Memorial Institute
S: sodium hydrosulfide
SCFAs: short‐chain fatty acids
SD: standard deviation
TGF‐β: transforming growth factor beta
TLR: toll‐like receptors
TNF‐α: tumor necrosis factor alpha
UV: ultraviolet
V: valerate

## 1 INTRODUCTION

Protein is an indispensable nutrient, being essential for cellular functions, connective tissue, and muscle synthesis.^1^ However, the average protein consumption in most western countries exceeds the recommended intake (0.8–1.0 g/kg of body weight per day).^2^ Despite most protein being broken down and absorbed in the upper gastrointestinal tract, a certain amount (~3 to 18 g/day)^3^, ^4^ of protein from diet and endogenous cells enters the colon and is fermented into various metabolites by the human gut microbes. In contrast to the short‐chain fatty acids (SCFAs) produced by fiber fermentation, protein fermentation in the distal colonic lumen contributes not only SCFAs, but also results in branched‐chain fatty acids (BCFAs), ammonia, hydrogen sulfide (H_2_S), p‐Cresol, and phenolic and indolic compounds.^5^ These metabolites influence the human host in different ways. For instance, it was shown that ammonium^6^, ^7^ and phenolic compounds^8^, ^9^, ^10^ are detrimental, whereas indolic compounds from tryptophan fermentation can attenuate inflammation through activation of aryl hydrocarbon receptor (AHR) signaling.^11^, ^12^ The exact influence of H_2_S is unclear as conflicting results were reported.^13^, ^14^ Besides, the BCFAs isobutyrate, 2‐methylbutyrate, and isovalerate are produced from the branched‐chain amino acids valine, leucine, and isoleucine, respectively^15^ and have not been associated with negative influences. As one of the SCFAs, microbial fermentation‐produced valerate has been reported to improve gut integrity at 2 mM^16^ and modulate lymphocytes^17^ and CD8^+^ T cell responses^18^ efficiently.

Dendritic cells (DCs) are antigen‐presenting cells, acting as the bridge between innate and adaptive immunity, where the cytokines produced in response to microbial stimuli induce local inflammation and determine the type of Th cells being activated.^19^, ^20^, ^21^, ^22^ For instance, interleukin‐6 (IL‐6) and tumor necrosis factor alpha (TNF‐α) are key pro‐inflammatory cytokines, with TNF‐α being produced for early immune response and IL‐6 contributing to both acute inflammation and the transition to adaptive immunity. IL‐1β, another pro‐inflammatory cytokine, is central in the activation of innate immunity and is able in itself to induce inflammation. The two cytokines, IL‐12 and IL‐23, share the p40 subunit and play pivotal roles in the differentiation and activation of Th1 and Th17 cells, respectively. Furthermore, Interferon beta (IFN‐β) serves as a key mediator of antiviral responses but is also induced by some intact bacteria and induces the transcription of many cytokines and defense proteins.^23^ IL‐10 and transforming growth factor beta (TGF‐β), on the other hand, are anti‐inflammatory cytokines essential for regulating excessive immune responses and inducing tolerance.^24^ Together, these cytokines reflect the interplay between pro‐ and anti‐inflammatory signals from DCs.

It is well‐established that SCFAs, especially butyrate, regulate the microbial‐induced immune response through multiple pathways, including G‐protein‐coupled receptors (GPRs) and histone deacetylase (HDACs) inhibition.^25^ Butyrate influences the differentiation and maturation of DCs and downregulates the IL‐12 production while increasing the production of IL‐23 in LPS‐stimulated DCs.^26^ Furthermore, long‐term treatment of DCs with butyrate inhibited the production of IL‐12.^27^ Compared to the widespread attention to the immune‐modulating effect of the SCFAs (butyrate, propionate, and acetate), the knowledge regarding the immune regulation by other gut microbial metabolites like protein fermentation‐derived products is limited. By assessing how the cytokine production is modulated by metabolites from protein fermentation in bacteria‐stimulated DCs, insights can be gained into the putative immunological effects of such protein breakdown products, in particular their impact on Th cell differentiation and other key immune cells. We hypothesized that metabolites derived from protein fermentation in the gut could modulate the immune response, with their specific effects varying depending on the type of microbial stimuli. Less‐studied SCFAs such as valerate, typically derived from protein fermentation, may exert regulatory effects as potent as butyrate.

In the present study, we selected 10 key by‐products of protein fermentation in the gut, including isobutyrate, 2‐methylbutyrate, isovalerate, valerate, ammonia, phenol, p‐Cresol, indole, and sulfides, encompassing BCFAs, aromatic compounds, and nitrogenous substances, and compared their impact on cytokine production using different microbial stimuli, including lipopolysaccharides (LPS), Ultraviolet (UV)‐inactivated *Lactobacillus acidophilus* NCFM, or *Staphylococcus aureus* USA300 in bone marrow‐derived dendritic cells (BMDCs). Due to the most prominent but varying effect of the four fatty acids, we studied their effect in more detail and compared the effects to the well‐described metabolite butyrate. We believe our results provide valuable information for understanding the potential influence of metabolites coming from proteinaceous substrates and serve as references for further in‐depth explorations.

## 2 MATERIALS AND METHODS

### 2.1 Protein/amino acid‐derived metabolites preparation

Ten compounds, including indole (reference number W259306), phenol (W322318), p‐Cresol (W233706), ammonium chloride (213330), sodium hydrosulfide (161527), iso‐butyrate (58360), 2‐methylbutyrate (193070), isovalerate (129542), valerate (240370), and sodium butyrate (303410), were purchased from Sigma‐Aldrich (St. Louis, MO, USA). The stock preparation was in sterile Milli‐Q water except indole, which was dissolved in ethanol (Sigma‐Aldrich, USA, reference number E7148). Final concentrations of ethanol never exceeded 0.1% on the cells. To exclude the impact of pH, NaOH (Sigma‐Aldrich, USA, reference number 567530) at 50 mM was used in stock preparation when needed. Stock solutions were further diluted with cell culture medium at desired concentrations for treatments.

### 2.2 Bacterial strains preparation

Two gram‐positive bacteria, *L. acidophilus* NCFM (Danisco, Copenhagen, Denmark) and clinical methicillin‐resistant *S. aureus* strain USA300, used in this study were prepared according to the previous report.^28^, ^29^ *L. acidophilus* NCFM grew anaerobically in de Man Rogosa Sharp broth (Merck, Darmstadt, Germany, product number 1.10661), whereas USA300 was streaked on tryptic soy agar (Merck, Darmstadt, Germany, product number 14432) and grown in tryptic soy broth (Merck, Darmstadt, Germany, product number 22092) at 37°C. Bacteria were washed twice in sterile phosphate‐buffered saline (PBS, Sigma‐Aldrich, USA, reference number D8537) and seeded in Petri dishes for colony‐forming unit (CFU) determination. UV pulsation (>90 s; 6 s/pulse with a monochromatic wavelength of 254 nm; CL‐1000 crosslinker; UVP, Cambridge, UK) was used to kill the bacteria, and the viability was verified afterward.

### 2.3 BMDCs generation, stimulations, and treatments

BMDCs were generated from C57BL/6NTac mice (Taconic, Lille Skensved, Denmark) as described previously.^30^, ^31^ Briefly, cells were isolated from the tibia and femur by flushing the bones with cold PBS, after which cells were seeded at 3 × 10^5^ cells/mL in Roswell Park Memorial Institute (RPMI) 1640 medium (Gibco™, USA, reference number 31870‐025) containing 10% (v/v) heat‐inactivated fetal bovine serum (FBS, Gibco™, USA, reference number 10270106), L‐glutamine (4 mM, Gibco™, USA, reference number 25030‐081), penicillin–streptomycin (100 U/mL, Gibco™, USA, reference number 15140‐122), and 2‐mercaptoethanol (50 μM, Gibco™, USA, reference number 31350‐010). On Days 3 and 6, culture medium containing 15 ng/mL granulocyte‐macrophage colony stimulating factors (GM‐CSF) was added to Petri dishes for DC differentiation. GM‐CSF was added as a culture supernatant harvested from a GM‐CSF‐producing cell line (a GM‐CSF transfected Ag8.653 myeloma cell line). Non‐adherent BMDCs were harvested on Day 8 and diluted into 2 × 10^6^ cells/mL, after which prepared compounds at desired concentrations were added for pre‐incubation for 30 min at 37°C in 5% CO_2_. LPS (0.1 μg/mL, *Escherichia coli* O26:B6, Sigma‐Aldrich, St Louis, MO, USA, reference number L2654), UV‐treated *L. acidophilus* NCFM (MOI 1), or *S. aureus* USA300 (MOI 10) was added to simulate inflammation afterward. For the long‐term treatment, BMDCs were pre‐treated with butyrate or valerate for 2 days prior to stimulation with LPS.

The Danish Animal Experimentation Act, LBK no. 474, approved the housing conditions for all mice used in the study to generate BMDCs. The Council of Europe Convention European Treaty Series (ETS)123 on the Protection of Vertebrate Animals Used for Experimental and Other Scientific Purposes' standards were followed when conducting the study.

### 2.4 BMDCs viability assay with flow cytometry

The effects of protein fermentation compounds on cell viability were assessed by calculating the intensity of the cellular fluorescence produced from 7‐aminoactinomycin D (7‐AAD, Invitrogen™, Carlsbad, CA, USA, reference number A1310) using a BD FACS Diva flow cytometer (BD Biosciences, Franklin Lakes, NJ, USA). In brief, cells were washed with cold PBS after 20 h of incubation and stained with 7‐AAD for 1–3 h, after which the dead cells were measured on a flow cytometer with the calculation of the mean fluorescent intensity (MFI) using the FlowJo™ software (version 10.6.2, BD Life Sciences, Ashland, OR, USA). All events were counted at 20,000/sample for the analysis.

### 2.5 Cytokine secretion assay

Cytokine production assay was performed in collected supernatants after 20 h of incubation. Murine cytokines including TNF‐α (catalog number DY410), IL‐6 (DY406), IL‐10 (DY417), IL‐12p70 (DY419), IL‐23 (DY1887), IFN‐β (DY8234‐05), TGF‐β (DY1679), and IL‐1β (DY401) were detected using a Duoset enzyme‐linked immunosorbent assay (ELISA, R&D Systems, Minneapolis, MN, USA) according to the manufacturer's manual.

### 2.6 Determination of intracellular reactive oxygen species formation

Reactive oxygen species (ROS) formation was determined by measuring the intensity of the cellular fluorescence produced from the oxidation of the internalized carboxy‐H_2_DCFDA (Invitrogen™, Carlsbad, CA, USA, reference number C400) on a BD FACS Diva flow cytometer (BD Biosciences, Franklin Lakes, NJ, USA). Briefly, murine BMDCs at 2 × 10^6^ cells/mL were stained with 5 μM carboxy‐H_2_DCFDA and pre‐incubated with prepared fatty acids (1 mM) at 37°C and 5% CO_2_ for 30 min following the addition of 0.1 μg/mL LPS. BMDCs treated with fatty acids or LPS only, as well as unstained BMDCs, were listed as controls. After another 4 h of incubation, the ROS formation was determined immediately, and the differences among fatty acids were assessed by calculating the mean MFI of treated BMDCs using the FlowJo™ software (version 10.6.2, BD Life Sciences, Ashland, OR, USA) with all events counted at 20,000/sample.

### 2.7 RNA extraction, cDNA synthesis, and quantitative real‐time polymerase chain reaction (qPCR)

RNA from BMDCs after treatments at different time points (2–8 h) was extracted using the MagMAX‐96 Total RNA Isolation Kit (Applied Biosystems, Foster City, CA, USA, reference number AM1830) according to the manufacturer's instructions. Genes *Ifnβ*, *Il10*, *Il12a, Il12b*, *Il23a*, *Ahr*, cytochrome p450 1a1 (*Cyp1a1)*, indoleamine 2,3‐dioxygenase 1 (*Ido1*), and dual specificity phosphatase 1 (*Dusp1*) were included in this study. cDNA was converted from RNA using High‐Capacity cDNA Reverse Transcription Kit (Applied Biosystems™, USA, reference number 4368814). Primers of the tested genes were listed in Table S1. Beta‐actine (*Actb)* was used as the reference gene to determine the ∆Ct of each sample (∆Ct_target_ = Ct_target_ − Ct_reference_). The fold gene expression of the samples was analyzed by subtracting the ∆Ct of the unstimulated cells (∆∆Ct = ∆Ct_target_ − ∆Ct_control_) followed by the 2^(−(∆∆Ct))^ method.

### 2.8 Statistics

All the experiments in this study were carried out with at least three biological replicates, including three technical repeats each time. Data are shown as means ± standard deviation (SD) and analyzed using GraphPad Prism software (version 9.3.1, GraphPad Software, San Diego, CA, USA). The differences of protein fermentation metabolites on cytokine production, cell viability, and ROS production were assessed by one‐way analysis of variance (ANOVA) with Dunnett as post‐test. Šídák's multiple comparisons test was applied in Figures 7A and 8 to see the differences from media or LPS, respectively, with no added fatty acids. Medium indicates unstimulated cells. *p < 0.05, **p < 0.01, ***p < 0.001, and ****p < 0.0001.

## 3 RESULTS

### 3.1 Metabolites of protein fermentation affect BMDC viability

**FIGURE 1:**
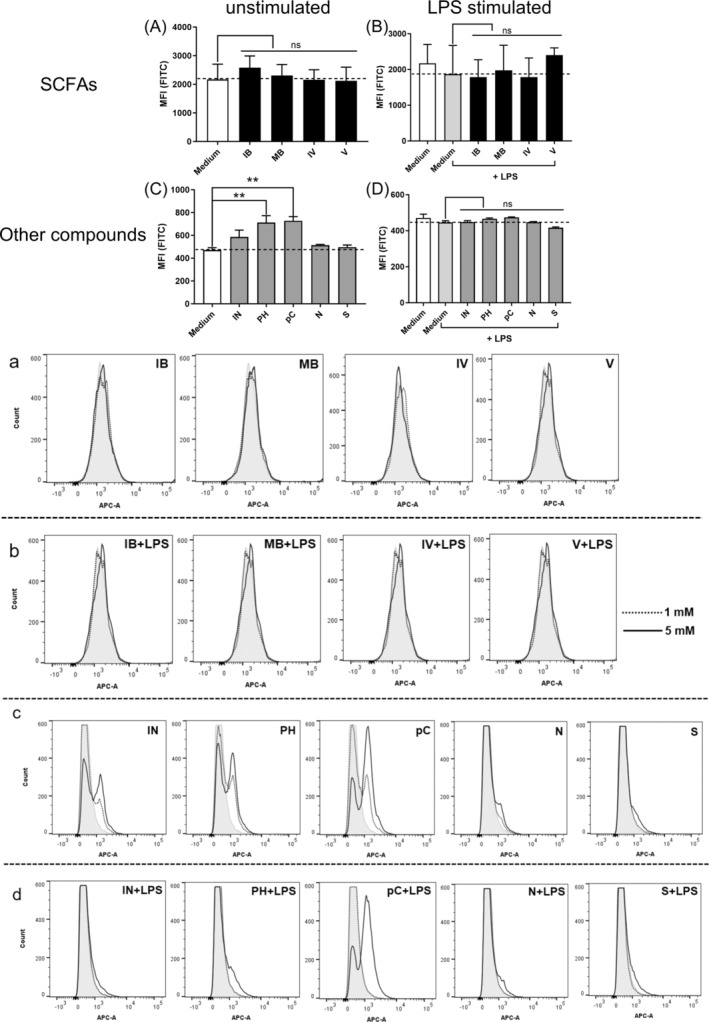
The influence of nine protein fermentation products on the viability of unstimulated or lipopolysaccharides (LPS)‐stimulated bone marrow‐derived dendritic cells (BMDCs). The compounds were added to BMDC 30 min prior to the stimulus LPS. After 20 h of incubation, viability was assessed by the addition of 7‐aminoactinomycin D (7‐AAD) by flow cytometry. The median fluorescence index (MFI) of cells incubated with the various compounds (1 mM) is shown in (A) and (C) (unstimulated) and (B) and (D) (LPS stimulated). Representative flow cytometry histograms (1 or 5 mM) are shown in (a–d). Differences in viability were assessed by one‐way analysis of variance followed by Dunnett (**p < 0.01, ns: not significant). Medium, unstimulated cells; IB, isobutyrate; IN, indole; IV, isovalerate; MB, 2‐methylbutyrate; N, ammonium chloride; pC, p‐Cresol; PH, phenol; S, sodium hydrosulfide; V, valerate.

We first assessed the effect of the compounds at concentrations of 1 or 5 mM on BMDC viability (Figure 1). None of the fatty acids (isobutyrate, 2‐methylbutyrate, isovalerate, and valerate) affected the BMDC viability (Figure 1A,B). In contrast, the rest of the compounds impacted the viability to varying degrees. Only phenol and p‐Cresol at 5 mM caused cytotoxicity in cells stimulated with LPS, but at 1 mM these compounds reduced cell viability when added to the immature BMDCs (Figure 1C). Also, indole reduced the viability of immature BMDCs to some extent.

### 3.2 Modulation of cytokine production by protein fermentation products depends on stimulatory agents (LPS, *L. acidophilus* NCFM, and *S. aureus* USA300)

**FIGURE 2:**
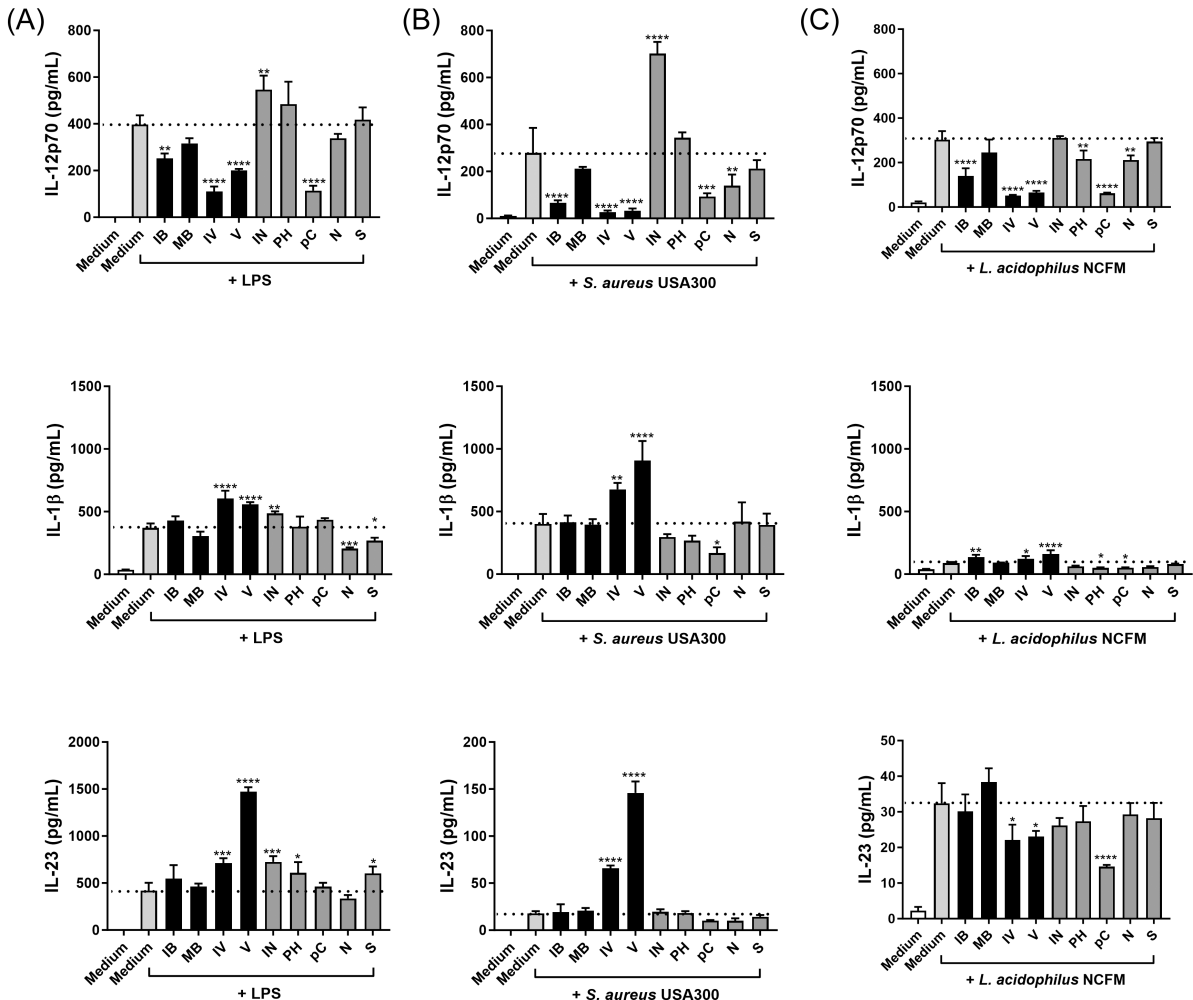
The effect of protein fermentation products on lipopolysaccharides (LPS) (A), *Staphylococcus aureus* USA300 (B), and *Lactobacillus acidophilus* NCFM (C) induced IL‐12p70, IL‐1β, and IL‐23 production. The compounds (1 mM) were added to bone marrow‐derived dendritic cells (BMDCs) 30 min prior to the bacterial stimuli. After 20 h of incubation, supernatants were harvested, and cytokine concentration was determined by ELISA after stimulation. Differences in the production of cytokine were assessed by one‐way analysis of variance followed by Dunnett (*p < 0.05, **p < 0.01, ***p < 0.001, ****p < 0.0001). *Y*‐axis coordinate range of IL‐23 is different among stimulations. Medium, unstimulated cells; IB, isobutyrate; IN, indole; IV, isovalerate; MB, 2‐methylbutyrate; N, ammonium chloride; pC, p‐Cresol; PH, phenol; S, sodium hydrosulfide; V, valerate.

When added to the BMDCs alone, none of the compounds induced the production of the cytokines investigated (results not shown). The effect of adding each of the compounds (1 mM) to the cells 30 min prior to microbial stimulation with LPS, *L. acidophilus* NCFM, or *S. aureus* USA300 on the production of IL‐12, IL‐1β, and IL‐23 is shown in Figure 2, and the TNF‐α, IL‐6, and IL‐10 production is shown in Figure S1. Differences in the cytokine production were in particular evident for the IL‐23, which was considerable after stimulation with LPS (>400 pg/mL) but low for *L. acidophilus* NCFM (<40 pg/mL), and of IL‐1β (from ~400 pg/mL after LPS or *S. aureus* USA300 stimulation to ~40 pg/mL after *L. acidophilus* NCFM stimulation).

When stimulated with *L. acidophilus* NCFM (panel C, Figures 2 and S1), p‐Cresol was the only metabolite that reduced the production of all cytokines. Isovalerate and valerate reduced IL‐12, IL‐23, and IL‐10 levels while increasing the production of IL‐1β. Other metabolites also affected the cytokine production, but to a lesser extent. When stimulated with *S. aureus* USA300 (panel B, Figures 2 and S1), p‐Cresol and ammonium chloride reduced the production of almost all cytokines, while indole enhanced the production of IL‐12 and TNF‐α, and fatty acids reduced the IL‐12, IL‐10, and IL‐6 production while enhancing the IL‐23 and IL‐1β (only isovalerate and valerate). Together with LPS stimulation (panel A, Figures 2 and S1), all metabolites reduced IL‐10 production, and some fatty acids further reduced IL‐12 and TNF‐α while enhancing IL‐23 and IL‐1β. Overall, the SCFAs exhibited the most varying and distinct effect on the bacteria‐induced cytokine production.

### 3.3 Compared to butyrate, some SCFAs show consistent and similar but weaker dose‐dependent effects on bacteria‐induced cytokines

**FIGURE 3:**
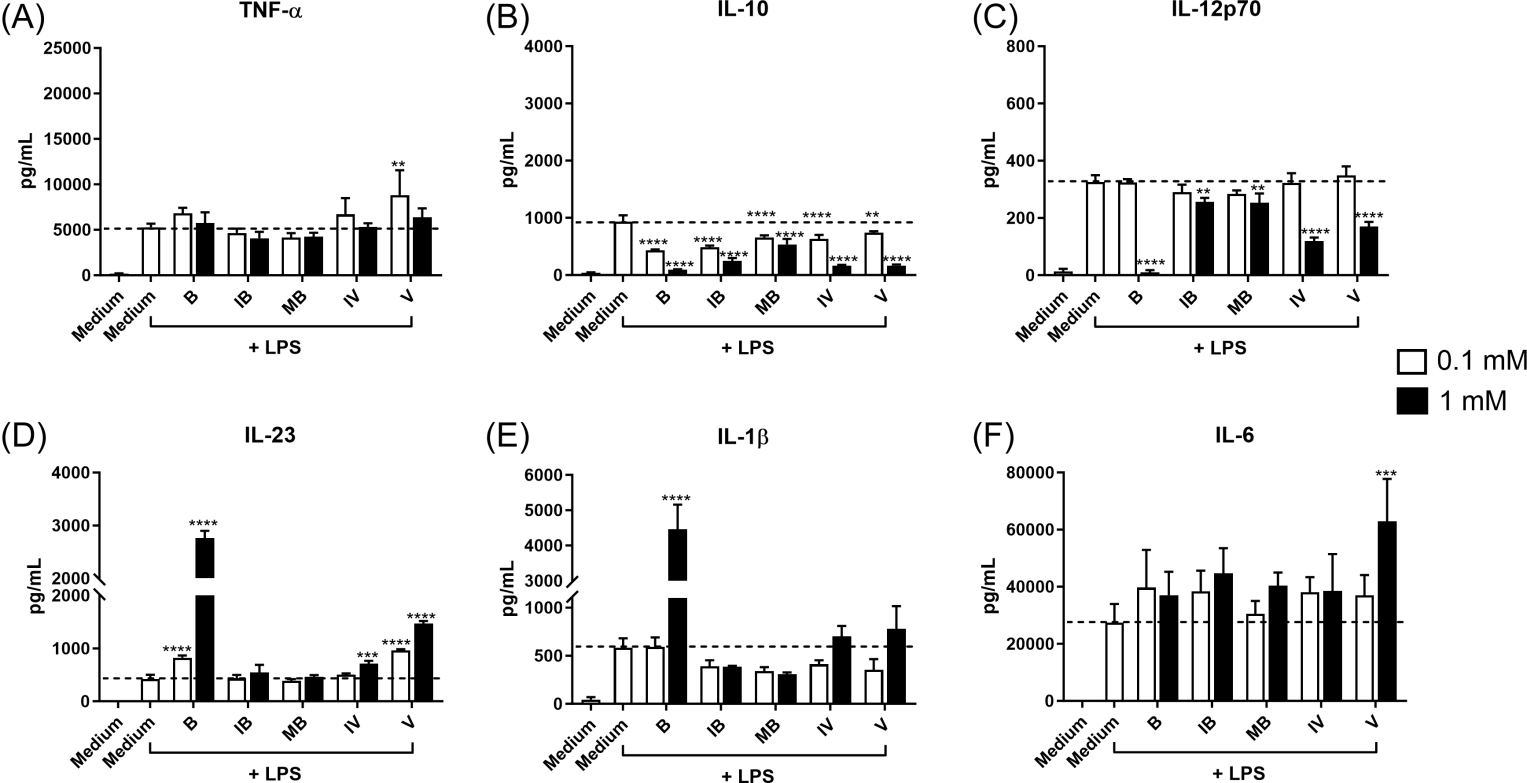
Immunomodulating dose‐dependent effect of various fatty acids on lipopolysaccharides (LPS)‐stimulated bone marrow‐derived dendritic cells (BMDCs) compared to butyrate. Different doses (1 mM vs. 0.1 mM) of fatty acid were added to BMDC 30 min prior to stimulation with LPS (0.1 μg/mL). Cytokine production, including TNF‐α (A), IL‐10 (B), IL‐12p70 (C), IL‐23 (D), IL‐1β (E), and IL‐6 (F), after 20 h was measured by ELISA. Differences in the production of cytokine were assessed by one‐way analysis of variance followed by Dunnett (**p < 0.01, ***p < 0.001, ****p < 0.0001). *Y*‐axis coordinate range of IL‐23 and IL‐1β is different among stimulations. Medium, unstimulated cells; B, butyrate; IB, isobutyrate; IV, isovalerate; MB, 2‐methylbutyrate; V, valerate.

**FIGURE 4:**
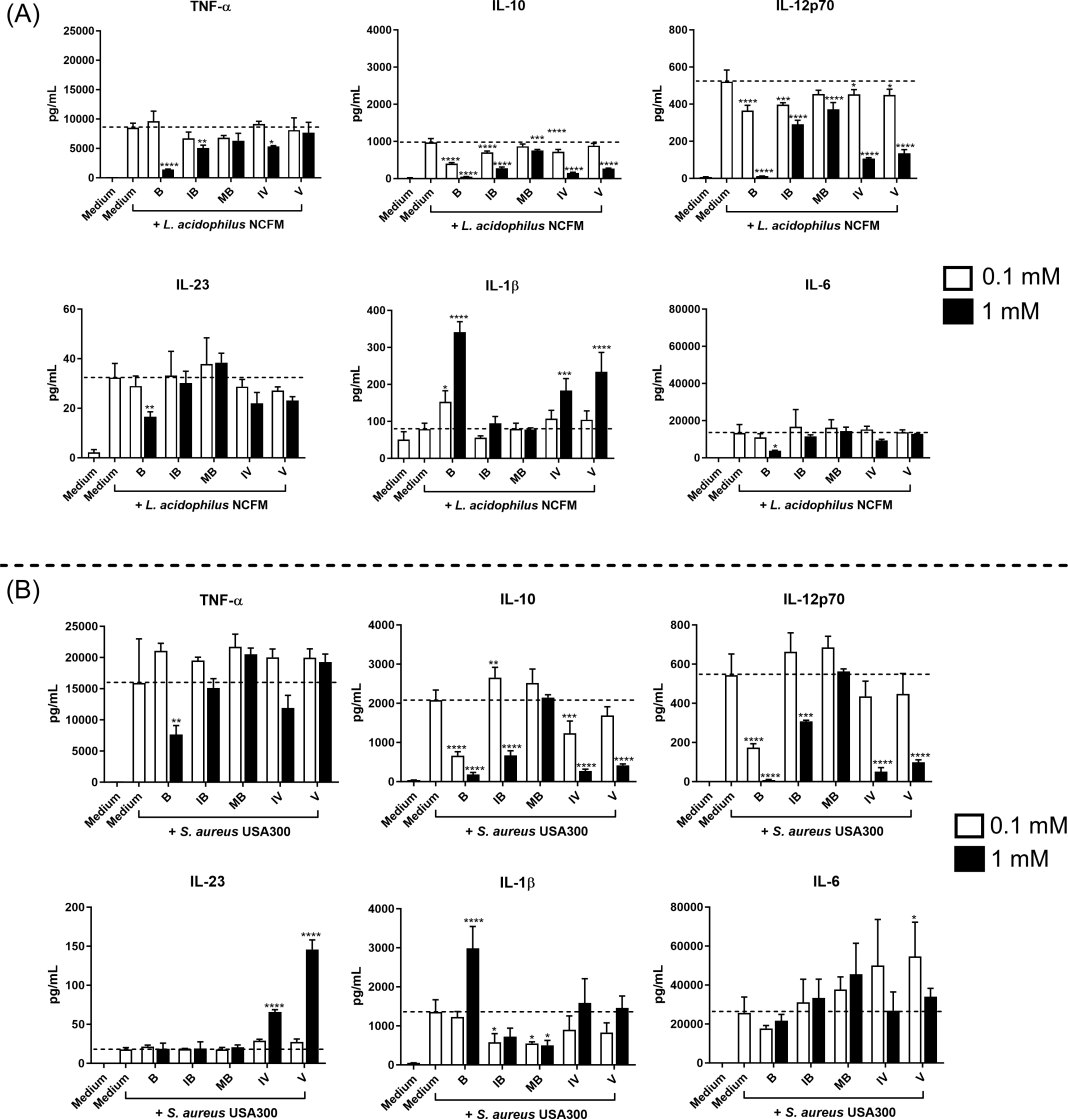
Immunomodulating dose‐dependent effect of various fatty acids on bacteria‐stimulated bone marrow‐derived dendritic cells (BMDCs) compared to butyrate. Different doses (1 mM vs. 0.1 mM) of fatty acid were added to BMDC 30 min prior to stimulation with *Lactobacillus acidophilus* NCFM (A) and *Staphylococcus aureus* USA300 (B). Cytokine production, including TNF‐α, IL‐10, IL‐12p70, IL‐23, IL‐1β, and IL‐6 after 20 h, was measured by ELISA. Differences in the production of cytokine were assessed by one‐way analysis of variance followed by Dunnett (* *p* < 0.05, **p < 0.01, ***p < 0.001, ****p < 0.0001). *Y*‐axis coordinate range of IL‐23 and IL‐1β is different among stimulations. Medium, unstimulated cells; B, butyrate; IB, isobutyrate; IV, isovalerate; MB, 2‐methylbutyrate; V, valerate.

We chose to assess the immunomodulating activity of the four SCFAs (isobutyrate, 2‐methylbutyrate, isovalerate, and valerate) compared to butyrate in two different concentrations, 0.1 and 1 mM. BMDCs were pre‐treated for 30 min with the fatty acids prior to stimulation with LPS (Figure 3), *L. acidophilus* NCFM, or *S. aureus* USA300 (Figure 4), and the production of cytokines was determined. Taken together, each fatty acid showed a consistent dose‐dependent but weaker influence on cytokine production compared to butyrate. Among the four fatty acids, valerate showed the strongest effect on modulating cytokine production.

At 0.1 mM, each fatty acid inhibited the LPS‐induced production of IL‐10, whereas only butyrate and valerate promoted IL‐23. All fatty acids at the high concentration of 1 mM reduced the LPS‐induced IL‐10 and IL‐12 production, and butyrate, isovalerate, and valerate promoted the production of IL‐23. Besides, butyrate and valerate at 1 mM led to high production of LPS‐induced IL‐1β and IL‐6, respectively (Figure 3).

When stimulated with *L. acidophilus* NCFM (Figure 4A), most fatty acids at 0.1 mM significantly decreased IL‐10 and IL‐12, while only butyrate at 0.1 mM increased the production of IL‐1β. At 1 mM, all fatty acids inhibited the production of IL‐10 and IL‐12, and some fatty acids decreased TNF‐α but increased IL‐1β. Butyrate was observed to have a downregulating effect on the production of TNF‐α, IL‐23, and IL‐6 at 1 mM. Upon *S. aureus* USA300 stimulation (Figure 4B), butyrate at 0.1 mM decreased IL‐10 and IL‐12, and isobutyrate promoted IL‐10 but decreased IL‐1β. Valerate at 0.1 mM increased *S. aureus* USA300‐induced IL‐6 production. At 1 mM, only butyrate inhibited TNF‐α, and most fatty acids, in particular isovalerate and valerate, decreased IL‐10 and IL‐12 but increased IL‐23 production.

Together, butyrate and valerate showed the strongest dose effect on modulating cytokine production among the fatty acids.

### 3.4 Valerate and butyrate have different effects on the ROS formation in BMDCs

**FIGURE 5:**
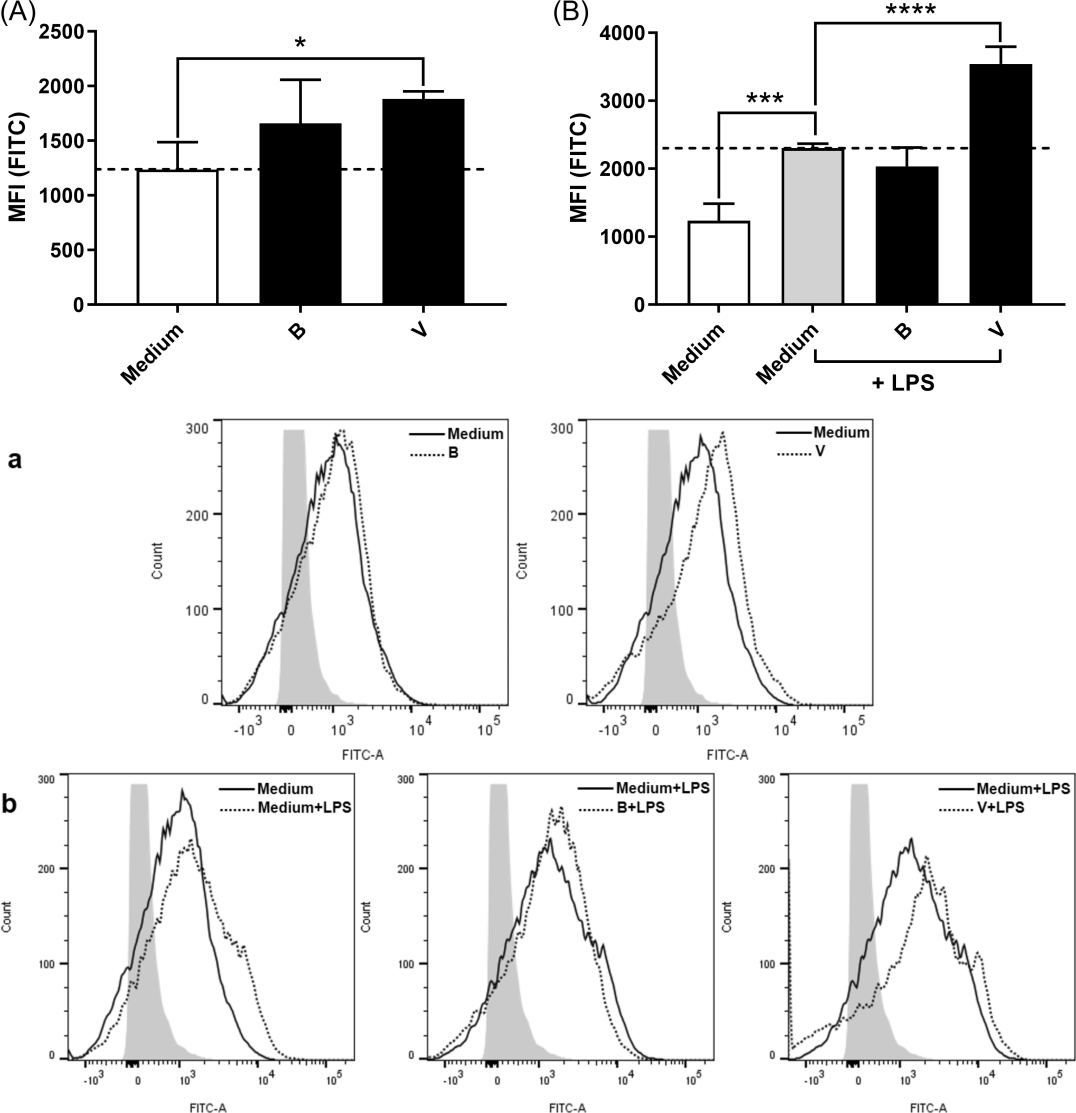
The influence of butyrate and valerate (1 mM) on reactive oxygen species (ROS) production with or without lipopolysaccharides (LPS) stimulation (0.1 μg/mL). Bone marrow‐derived dendritic cells (BMDCs) were pre‐treated for 30 min with butyrate or valerate prior to stimulation with LPS, and the ROS formation was measured after 4 h as the oxidation of carboxy‐H_2_DCFDA by flow cytometry. The mean (*n* = 3) fluorescence index (MFI) is shown in (A and B), and representative flow cytometry results are shown in (a and b). Differences in MFI were assessed by one‐way analysis of variance followed by Dunnett (*p < 0.05, ***p < 0.001, ****p < 0.0001). Medium, unstimulated cells; B, butyrate; V, valerate.

The effect of adding butyrate or valerate to the cells 30 min prior to LPS stimulation on the ROS formation as determined by the oxidation of carboxy‐H_2_DCFDA is shown in Figure 5. In non‐stimulated cells, only valerate showed an influence on the ROS formation of BMDCs. After LPS stimulation, the ROS production increased as expected^32^ and was further increased by the addition of valerate but not butyrate (Figure 5B,b).

### 3.5 Butyrate affects cytokine‐encoding gene expression more promptly than valerate

**FIGURE 6:**
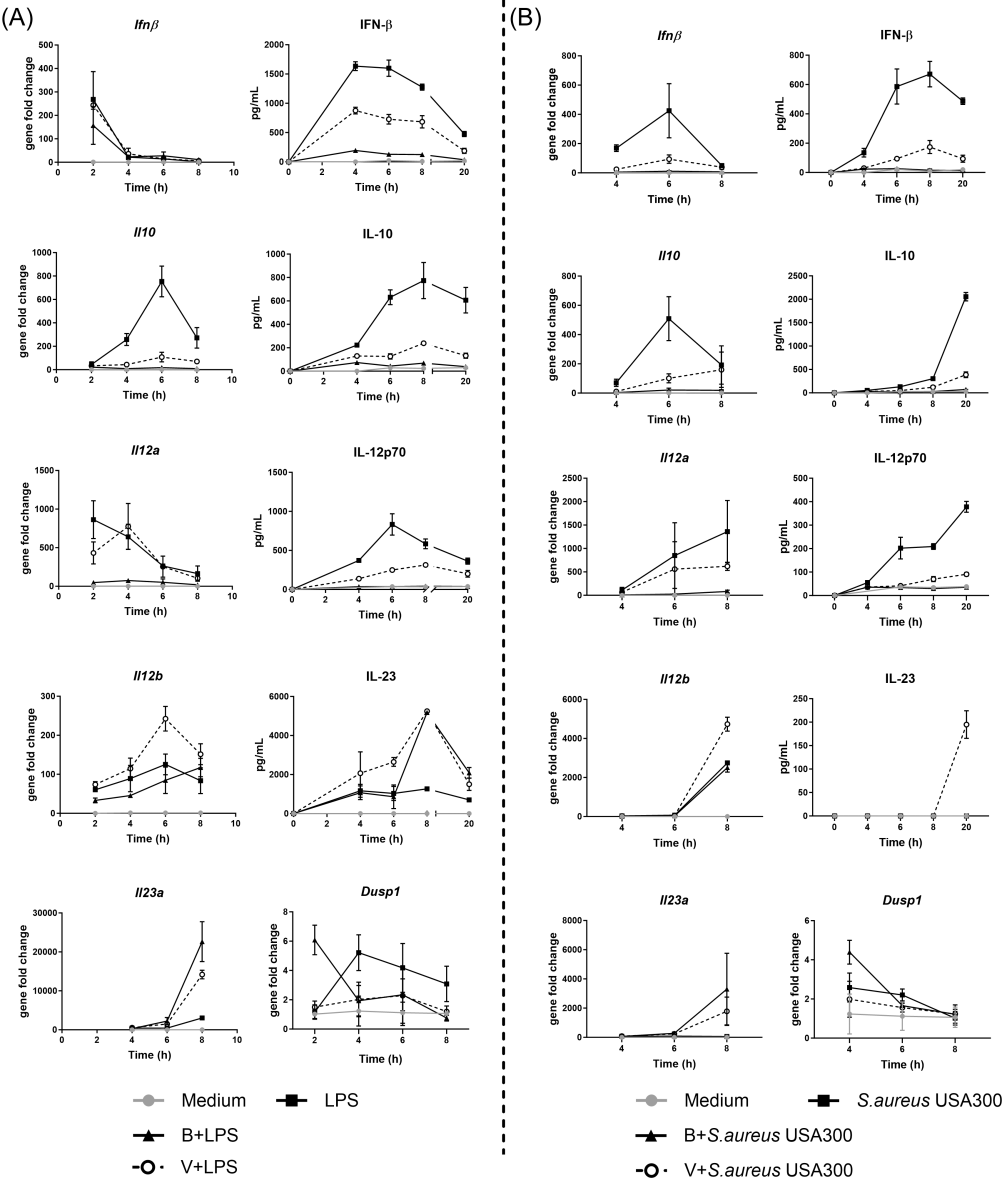
The influence of butyrate and valerate on the expression kinetics of the cytokines (IFN‐β, IL‐10, IL‐12p70, and IL‐23) and the encoding genes (*Ifnβ*, *Il10*, *Il12a*, *Il12b*, *Il23a*, and *Dusp1*) in bone marrow‐derived dendritic cells (BMDCs) stimulated with lipopolysaccharides (LPS) (A) or *Staphylococcus aureus* USA300 (B). BMDCs were pre‐treated for 30 min prior to stimulation with LPS or *S. aureus* USA300. Medium, unstimulated cells; B, butyrate; V, valerate.

To assess how butyrate and valerate affected the expression kinetics of the cytokines IFN‐β, IL‐10, IL‐12, and IL‐23, BMDCs were pretreated with butyrate or valerate for 30 min prior to stimulation with LPS or *S. aureus* USA300, and the expression of genes *Ifnβ*, *Il10*, *Il12a*, *Il12b*, and *Il23a* encoding for the respective cytokines, as well as the *Dusp1*, were measured after 2 (only LPS stimulation), 4, 6, and 8 h, and the cytokines in the supernatant were quantified (Figure 6). Cells treated with LPS gave a more prompt response relative to *S. aureus* USA300 stimulation, as reflected by the very early increase in *Ifnβ* and *Il12a* expression peaking after 2 h compared to 6 h or later after *S. aureus* USA300 stimulation. In contrast, the expression of *Il10* and *Il23a* peaked later, after 6 and 8 h, respectively, with no difference between the two stimuli. Addition of butyrate resulted in an almost complete abrogation of both LPS and *S. aureus* USA300‐induced IFN‐β, IL‐12, and IL‐10 production. In contrast, valerate only led to a partial reduction in IFN‐β and IL‐12 production, especially after LPS stimulation, where the suppression was only modest. The different effects of butyrate and valerate on the LPS and *S. aureus* USA300‐induced IFN‐β and IL‐12 and corresponding genes were especially evident at the early time points after stimulation.

One key mechanism in the abrogation of the microbially stimulated IFN‐β and IL‐12 in BMDCs is increased de novo synthesis of *Dusp1* that efficiently dephosphorylates JNK and P38.^33^, ^34^, ^35^ Strikingly, the expression of *Dusp1* was promptly upregulated after butyrate but not after valerate treatment.

### 3.6 Long‐term pre‐treatment of BMDCs with butyrate or valerate inhibits IL‐12 and enhances IL‐10 and TGF‐β production

**FIGURE 7:**
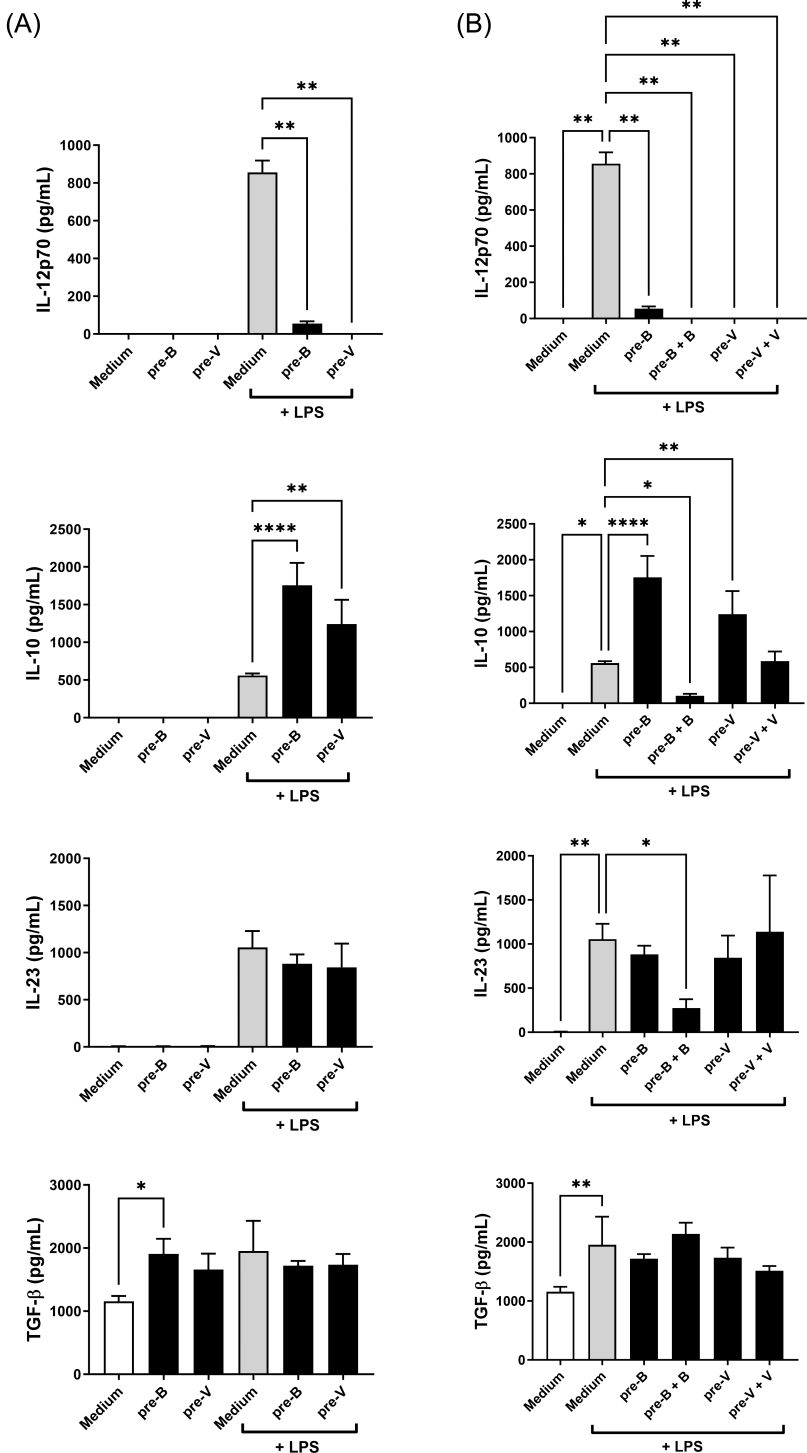
The production of cytokines in bone marrow‐derived dendritic cells (BMDCs) pre‐treated with butyrate (pre‐B) or valerate (pre‐V) for 2 days prior to harvest of cells and stimulation with lipopolysaccharides (LPS) (A) and when further added butyrate (B) or valerate (V) 30 min before stimulation with LPS (B). Cells were incubated for 20 h, and supernatants were harvested and analyzed for cytokine concentrations. Statistics: One‐way analysis of variance with multiple comparisons. (A): Šídák's multiple comparisons test. Asterisks show differences from media or LPS, respectively, with no added fatty acids. (B): Dunnett's multiple comparisons test. Asterisks show differences between LPS‐stimulated cells with no added fatty acids. *p < 0.05, **p < 0.01, ****p < 0.0001. Medium, unstimulated cells.

As cells may be conditioned by dietary fermentation metabolites long in advance to microbial stimulation, we aimed to assess the effect of adding butyrate or valerate 2 days before the fully differentiated DCs were harvested (pre‐treatment) and compare this treatment with the effect of adding valerate or butyrate 30 min before stimulation with LPS (Figure 7). Expectedly, pre‐treatment of the cells with either butyrate or valerate without any microbial stimuli did not give rise to the production of IL‐12, IL‐10, or IL‐23. However, we also quantified TGF‐β, which is constitutively expressed by immature DCs, and TGF‐β raised to the level seen after stimulation with LPS (Figure 7A). When butyrate or valerate was added 2 days before stimulation with LPS, the induced IL‐12 was almost completely abrogated, IL‐10 was further increased, while the IL‐23 and TGF‐β production was unaffected (Figure 7A). Together, this indicates that both butyrate and valerate added during the late period of the DC development imprint a tolerogenic phenotype. When adding butyrate or valerate just before stimulation with LPS to cells pretreated with butyrate or valerate, butyrate strongly suppressed the induced IL‐10 and IL‐23, while valerate only reduced IL‐10 and only to the level induced by LPS (Figure 7B). Adding valerate to cells pretreated with butyrate (Figure S2) and stimulating with LPS resulted in cytokine production comparable with cells pre‐treated with valerate, thus further demonstrating that the effects of pre‐treatment with the two SCFAs were comparable, while the short‐term effects were markedly different.

**FIGURE 8:**
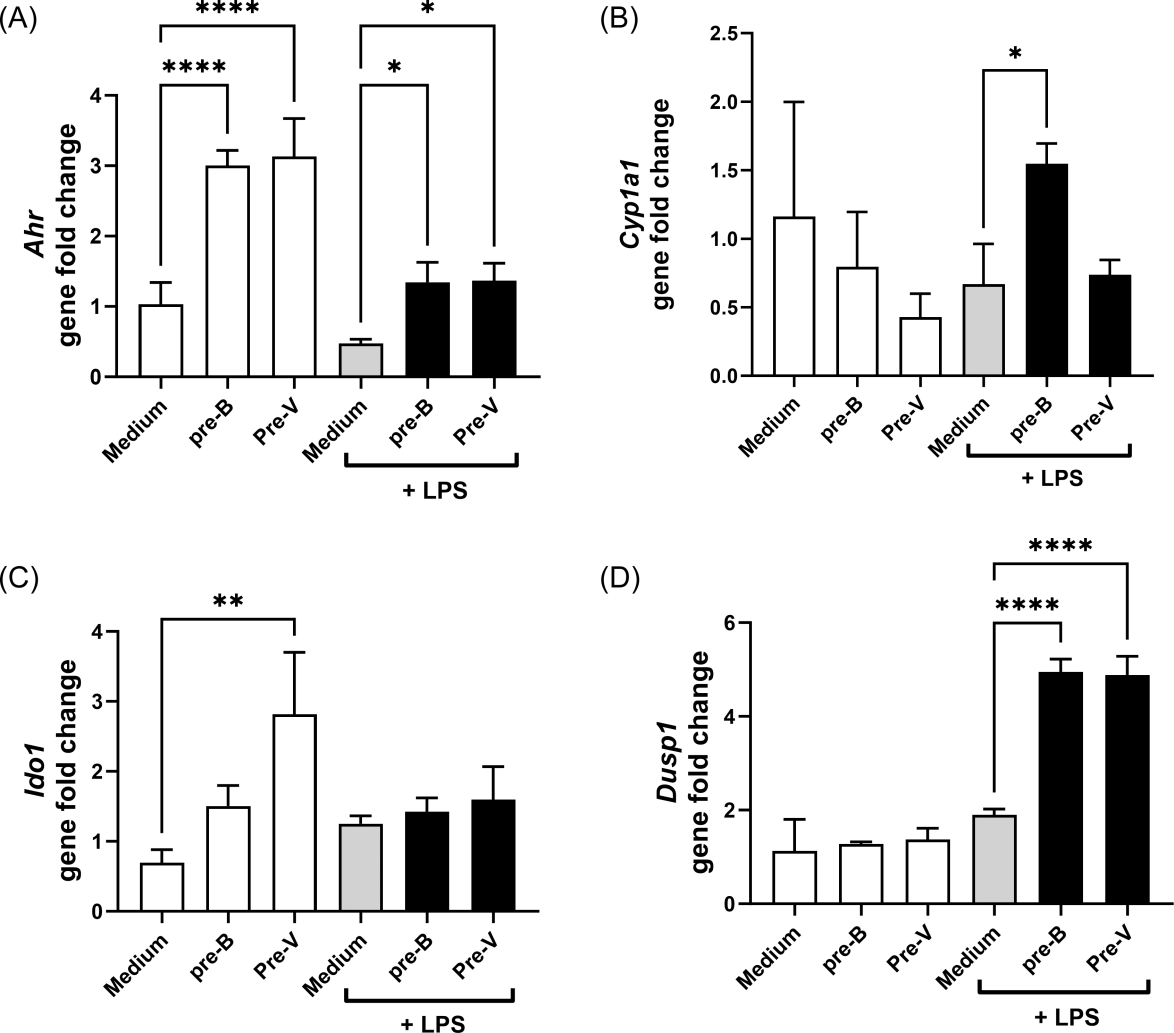
The expression of *Ahr* (A), *Cyp1a1* (B), *Ido1* (C), and *Dusp1* (D) in bone marrow‐derived dendritic cells (BMDCs) pre‐treated with butyrate (pre‐B) or valerate (pre‐V) for 2 days prior to stimulation with lipopolysaccharides (LPS) or media and incubated for 8 h before cell harvest and isolation of mRNA. Differences in gene fold change were assessed by one‐way analysis of variance with Šídák's multiple comparisons. Asterisks show differences from media or LPS, respectively. *p < 0.05, **p < 0.01, ****p < 0.0001. Medium, unstimulated cells.

A number of genes have previously been demonstrated to be involved in the regulation of microbial‐induced cytokine production and function of DCs, including *Ahr*,^36^ *Cyp1a1*,^37^ *Ido1*,^38^ and *Dusp1*. AHR is indispensable for the induction of IL‐10, which in turn inhibits the production of pro‐inflammatory cytokines.^39^, ^40^ Long‐term treatment with both butyrate and valerate enhanced the expression of *Ahr* in the immature BMDCs, while LPS reduced the level of *Ahr* expression (Figure 8A). The LPS‐induced expression of IL‐10 after long‐term treatment (Figure 7A) corresponded with the increased *Ahr* expression. This pattern was also reflected in the expression of *Cyp1a1* upon butyrate treatment, although gene expression was weak 8 h after LPS stimulation (Figure 8B). Furthermore, pre‐treatment of the cells for 2 days led to an increase in the *Ido1* expression in non‐stimulated cells but not in LPS‐stimulated cells (Figure 8C). In contrast, the expression of *Dusp1* (Figure 8D) was upregulated in LPS‐stimulated cells but not in unstimulated cells upon pre‐treatment with the fatty acids.

## 4 DISCUSSION

A small but significant part of ingested proteins reaches the large colon undigested and may here become microbially fermented with the amino acids being converted into various metabolites by gut microbes. Compared to dietary fiber fermentation resulting in high production of SCFAs like butyrate, which has barrier‐enhancing and anti‐inflammatory functions,^41^, ^42^, ^43^ the knowledge regarding other metabolites from protein fermentation is scarce, with only the changes in epithelial integrity previously reported.^4^, ^44^, ^45^ Notably, gut‐derived metabolites enter systemic circulation, reaching distal organs and tissues where they can influence physiological processes and health.^46^ Elucidating the interactions between microbiota‐derived metabolites and the host can pave the way for therapeutic strategies applied to human microbiome‐related disorders. Here, we examined the potential effect of nine of these metabolites (SCFAs/BCFAs, indole, phenol, p‐Cresol, ammonium, and H_2_S) regarding their toxicity and their ability to modulate the induction of cytokine via various microbial stimuli (LPS, *L. acidophilus* NCFM or *S. aureus* USA300) in BMDCs. Of these compounds, the investigated SCFAs (isobutyrate, 2‐methylbutyrate, valerate, and isovalerate) exhibited low toxicity and the strongest immunomodulatory activity. We therefore investigated their effect on the BMDCs in more detail to assess the putative beneficial effect and found interesting differences in the modulating mechanisms. In specific, butyrate and valerate stood out as compounds inducing a tolerogenic phenotype in BMDCs.

Immune‐modulating effects describe how specific metabolites influence immune cell responses, distinct from toxic effects, which primarily result in cell damage or death. Metabolites from the colonic fermentation are known to exhibit significant inter‐ and intra‐individual variation and present across a wide concentration range, as evidenced by butyrate concentrations in the range of 0.04–5.96 μmol/g and valerate of 0.33–4.10 μmol/g.^47^, ^48^, ^49^ Here, we chose the concentrations of 1 and 5 mM to test the influence of these compounds on the viability of DCs and applied 1 mM and below for further investigation of their immune‐modulating activity. Among the metabolites investigated, SCFAs demonstrated significant immune modulation without markedly impacting cell viability, suggesting that their influence operates through signaling pathways rather than cytotoxic mechanisms. The rest of the compounds affected the viability of immature BMDCs, but to a varying degree. p‐Cresol in particular exhibited high toxicity, both when added to immature and to LPS‐stimulated BMDCs, and this correlated with a reduced cytokine production for all cytokines upon p‐Cresol addition, thus indicating that the toxic effect influenced the cytokine production. The harmful effects of p‐Cresol^50^ and phenol^8^ were also reported in previous studies on colonic epithelial cells, where a concentration of 1 mM of phenolic compounds strongly impacted the viability. When assessing the effect on the cell viability in LPS‐stimulated (mature) DCs, the toxic effect of p‐Cresol was still high, indicating a continuous killing of cells during or after maturation. This contrasts with the effect of many of the other compounds, which showed low proportions of dead cells after the addition of LPS. This may indicate that maturation by LPS, which induces increased endocytotic activity,^51^ could facilitate the clearing of dead cells through endocytosis. The effect on mature cells by p‐Cresol indicates that this compound prevents (fully or partly) maturation by LPS. We did not, however, investigate this further. Indole‐treated cells revealed only minor toxicity but nevertheless significantly reduced the microbially induced production of IL‐10 and enhanced the *S. aureus* USA300 and LPS‐induced IL‐12. Indole and its derivatives are derived from the metabolism of tryptophan by gut microorganisms.^52^ Here we only tested indole and not the various derivatives, which may exert different strengths and effects.

Our data do not allow us to distinguish between effects caused by cell toxicity and effects caused by changes in cell signaling. Accordingly, we chose to focus the study on SCFAs, including some BCFAs, which all demonstrated no or very low toxicity. It is well‐established that different microbial stimulations cause differences in the resulting cytokine production by DCs due to activation of distinct signaling pathways in the cell,^20^, ^53^ but it is not clear to which extent the effects of the metabolites depend on the microbial signal. In contrast to the other metabolites investigated, the tested SCFAs exhibited quite varying effects in the differently stimulated cells. Especially for the production of IL‐23, TNF‐α, and IL‐6, we observed marked differences depending on the microbial stimuli. These findings indicate that SCFAs have fewer side effects and higher capacities in modulating cytokine production in comparison to other types of metabolites arising from protein fermentation. We have previously shown similar dependences on the microbial signal for the carbohydrates mannan and β‐glucan^28^ as well as for stilbenoids.^29^ Hence, the type of stimulation should be considered when assessing the immunomodulatory effect of SCFAs *in vitro*.

Comparing two different doses of SCFAs with the effect of the same doses of butyrate revealed that independently of the applied microbial stimuli, butyrate stood out as the SCFA with the strongest impact on the cytokine production. Isobutyrate, isovalerate, and especially valerate also exhibited potent immunomodulation while 2‐methylbutyrate was poor, indicating the importance of flexibility in the aliphatic chain close to the α‐carbon for the activity of the SCFAs. The distinct dose‐dependent effect of butyrate and valerate further underscores the benefits of dietary fiber fermentation in the colon compared to protein fermentation. One difference between the immunomodulating effects of the SCFAs seems to depend on the applied microbial stimuli. The IL‐23 production increased dramatically in butyrate‐treated cells after LPS stimulation, while after *S. aureus* USA300, but not *L. acidophilus* NCFM stimulation, valerate (and isovalerate) treatment revealed potent IL‐23 increasing capacity. The enhanced IL‐23 production seen after the addition of butyrate to LPS‐stimulated cells has been reported previously,^26^ but the lack of enhanced IL‐23 production after stimulation with whole bacteria has, to our knowledge, not been described before. An important difference between stimulation with LPS and the intact gram‐positive bacteria is that whereas LPS can induce an intracellular signaling cascade promptly by ligating to TLR4,^54^ the gram‐positive bacteria must be taken up and degraded endosomally in order to release Toll‐like receptors (TLR)‐activating ligands,^22^ a process that results in a markedly delayed response as seen by the late upregulation of cytokine gene transcription. Thus, depending on whether the metabolites affect the cells promptly through receptor binding and subsequent intracellular signaling modification or, more slowly, through epigenetic modification, this may affect the cytokine production differently. To this end, butyrate, but not valerate, showed prompt upregulation of *Dusp1* expression encoding the dual specificity phosphatase 1, which deactivates the MAP kinases p38 and JNK.^55^ We have shown that induction of the production of IL‐12 and IFN‐β depends on p38, JNK, or both kinases.^20^ Thus, butyrate may, through the early *Dusp1* upregulation, inhibit the early transcription of IL‐12 and IFN‐β encoding genes. As the induction of IFN‐β by *S. aureus* USA300 is slower, the early upregulation of *Dusp1* by butyrate may therefore have less impact on the response.

Endosomal ROS production is a prerequisite for the degradation of endocytosed proteins and phagocytosed intact bacteria like *S. aureus* USA300 to produce cytokines.^56^, ^57^ The increased ROS formation induced by valerate but not by butyrate (with or without LPS stimulation) may be a factor involved in the divergent cytokine responses seen for the two SCFAs. However, as we did not determine the source of ROS, we cannot know whether the increased ROS induced by valerate is induced through the activation of nicotinamide adenine dinucleotide phosphate (NADPH) oxidase or by an effect of valerate on the mitochondrial ROS production, as fatty acids have been demonstrated to have the capacity to affect both depending on the fatty acid as well as the cell type.^58^ Butyrate reportedly causes intracellular potassium ion outflow and hyperpolarization and calcium ion inflow, leading to activation of the NLRP3 inflammasome upon binding to GPR43 or GPR109a,^59^ which leads to increased IL‐1β production. With no known receptor for valerate, an alternative explanation for the increased IL‐1β could be the increased ROS production by valerate, as ROS is a known inducer of the NRLP3 inflammasome.^60^ This, however, is purely speculative and needs further investigation.

In contrast to the short‐term effect assessed when adding the metabolites 30 min prior to microbial stimulation, long‐term (2 days) treatment of the cells revealed comparable effects of butyrate and valerate on the cytokine production and was different from short‐term treatment, resulting in an upregulation of the IL‐10 response in the LPS‐stimulated BMDCs. Together, these differences between short‐ and long‐term treatment may indicate a difference in whether there is an involvement of receptor‐ligand interaction, such as the well‐described interaction between GPR109a and butyrate in DCs,^61^ or not. Long‐term treatment with both butyrate and valerate led to an increase in TGF‐β corresponding to the increase seen after LPS stimulation (around 50% increase) (without per se inducing the production of other cytokines) and increased expression of *Ahr* and *Ido1* expression. The transcription factor AHR is involved in the induction of IL‐10 upon LPS stimulation, downregulating the production of pro‐inflammatory cytokines^62^ and it further mediates the expression *of Ido1*.^63^ It has previously been demonstrated that in a microenvironment dominated by immunoregulatory TGF‐β, IDO‐1 becomes phosphorylated, which in turn leads to further upregulation of the expression of *Ido1* and *Tgfb1* genes,^64^ ultimately establishing a long‐term immunoregulatory DC phenotype. In contrast to *Ido1* and *Ahr*, the expression of *Dusp1* was increased only upon LPS stimulation, underscoring its effect in newly stimulated cells. This is confirmed by the changed effect of LPS stimulation after both butyrate and valerate long‐term treatment, leading to increased IL‐10 as opposed to short‐term treatment with butyrate showing the most prominent effect leading to diminished IL‐10 production.

## 5 CONCLUSION

In summary, this study found that unbranched SCFAs exerted significant immunomodulatory effects in DCs compared to other protein fermentation metabolites, with effects that vary based on the type of microbial signal but, in general, reduced the pro‐inflammatory response and upregulated the tolerance‐inducing cytokines. The comparatively weaker ability of valerate compared to butyrate to modulate cytokine production (IL‐12p70, IL‐10, IL‐23, and IL‐1β) after short‐term treatments, alongside with the tolerogenic DC phenotype (decrease in IL‐12 and enhancement of IL‐10) induced by both fatty acids, reveals both shared and distinct mechanisms through which these SCFAs influence cytokine production in BMDCs. These findings underscore the specific, nuanced ways in which protein fermentation metabolites modulate immune responses. In contrast to the major metabolites SCFAs produced in the colon, other metabolites such as BCFAs, sulfide, phenol, and indole products generated during protein fermentation exhibit no or even detrimental effects on DCs.

## AUTHOR CONTRIBUTIONS

ZX, DSN, and HF formulated the idea; ZX and HF designed this study; ZX carried out most of the experiments and analyzed the data; DBE and PRJ helped and performed parts of some experiments; ZX and HF wrote the manuscript; ZX, PRJ, DBE, DSN, and HF discussed and interpreted data; all authors critically revised and approved the final version of the manuscript.

## CONFLICT OF INTEREST STATEMENT

The authors declare no conflicts of interest.

## Supporting information


**Data S1.** Supporting Information.

## Data Availability

The data that support the findings of this study are available from the corresponding author upon reasonable request.
