# Supplementary material for: The effect of microbial metabolites from colonic protein fermentation on bacteria‐induced cytokine production in dendritic cells
Source: Biofactors. 2025 Feb 24;51(1):e70007. doi: 10.1002/biof.70007 (PMC11849446; doi:10.1002/biof.70007)
Supplement: Supplementary file 1 — Data S1. Supporting Information. [file BIOF-51-0-s001.docx]

**Supplementary table 1:** Primer sequences used for qRT-PCR analysis.

| **Gene** | **TaqMan™ Assay ID** | | **Sequence 5’to 3’** |
| --- | --- | --- | --- |
| *Actb* | Mm00607939_s1 | | tttacaccctttctttgacaaaacctaacttgcgcagaaaaaaaaaaaataagagacaacattggcatggctttgtttttgtttttttaatttttttttaaagtttttttttttt |
| *Ifnβ1* | Mm00439552_s1 | | ctgggactggtagtgaatctactgcatttgaaaggtcaaaggaaaacagagtttttattaatttataat |
| *Il12a* | Mm00434165_m1 | | cggccagagaaaaactgaaacattattcctgcactgctgaagacatcgatcatgaagacatcacacgg |
| *Il12b* | Mm00434174_m1 | | catcaaaccagacccgcccaagaacttgcagatgaagcctttgaagaactcacaggtggaggtcagctgggagta |
| *Il23a* | Mm00518984_m1 | | ttctgcttgcaaaggatccgccaaggtctggctttttataagcacctgcttgactctgaca |
| *Il10* | Mm01288386_m1 | | caagaccaaggtgtctacaaggccatgaatgaatttgacatcttcatcaactgcatagaagcatacatgatgatcaaaatgaaaagctaaaacacctgcagtgtgtattgagtctgctggactccaggacctagac |
| *Ahr* | Mm00478932_m1 | | tacaggcgctgaatggctttgtgctggttgtcacagcagatgccttggtcttctatgcttc |
| *Ido1* | Mm00492586_m1 | | tggagctgcccgacgcatacagcccctgggtccttgtggctagaaatctgcctgtgctgattgagaacgggcagcttcgagaa |
| *Dusp1* | Mm00457274_g1 | | ggcccagtggagatcctgtccttcctgtacctgggcagtgcctatcacgcttctcggaaggatat |
| *Cyp1a1* | | Mm00487218_m1 | gaagggccacatccgggacatcacagacagcctcattgagcattgtcaggacaggaagctggacgagaatgccaatgtccagctgtcagatgataaggtcatcacgattgttttggacct |

**Supplementary figure 1**: The effect of protein fermentation products on LPS **(A)**, *S. aureus* USA300 **(B)**, and *L. acidophilus* NCFM **(C)** induced TNF-α, IL-6, and IL-10 production. The compounds (1mM) were added to BMDC 30 min prior to the bacterial stimuli. After 20 h incubation supernatants were harvested and cytokine concentration was determined by ELISA after stimulation. Differences in the production of cytokine were assessed by one-way analysis of variance (ANOVA) followed by Dunnett (* p < 0.05, ** p < 0.01, *** p < 0.001, **** p < 0.0001). IB, isobutyrate; MB, 2-methylbutyrate; IV, isovalerate; V, valerate; IN, indole; PH, phenol; pC, p-Cresol; N, ammonium chloride; S, sodium hydrosulfide.

**Supplementary figure 2**: The production of cytokines in BMDCs pre-treated with butyrate (pre-B) for 2 days prior to harvest of cells and when further added butyrate (B) or valerate (V) 30 min before stimulation with medium **(A)** or LPS **(B)**. Cells were incubated for 20 h and supernatants were harvested and analyzed for cytokine concentrations. Statistics: Dunnett's multiple comparisons test. Asterisks show differences between LPS-stimulated cells with no added fatty acids. * p < 0.05, ** p < 0.01, *** p < 0.001, **** p < 0.0001.
